# Chrono‐Synergistic Nutrition: Gut Microbiota–Targeted Diets and Circadian‐Aligned First Meal Timing Confer Robust Protection Against Kidney Stone Formation

**DOI:** 10.1002/fsn3.71297

**Published:** 2025-12-15

**Authors:** Tianxiang Fu, Xuanhao Zhou, Zhihong Chen, Zhuoyan Huang, Jingxian Li, Qiuyang Li, Wenqi Wu, Yiming Tang

**Affiliations:** ^1^ Department of Urology, The Second Affiliated Hospital Guangzhou Medical University Guangzhou Guangdong People's Republic of China; ^2^ Second Clinical College Guangzhou Medical University Guangzhou Guangdong People's Republic of China; ^3^ Department of Obstetrics & Gynecology, Third Affiliated Hospital Sun Yat‐Sen University Guangzhou Guangdong People's Republic of China

**Keywords:** chrono‐nutrition, dietary index for gut microbiota, first meal timing, gut‐kidney axis, kidney stones, NHANES

## Abstract

The emerging gut–kidney axis paradigm suggests that dietary modulation of gut microbiota may influence nephrolithiasis pathogenesis, while the specific dietary components and the temporal dimension of nutritional intake remain unexplored. This study introduces a novel framework integrating gut microbiota‐targeted dietary quality with circadian‐aligned meal timing to elucidate their synergistic role in kidney stone prevention. We conducted a comprehensive analysis of 21,840 adults from the NHANES cohort (2007–2016), investigating the independent and combined associations of the Dietary Index for Gut Microbiota (DI‐GM) and First Meal Timing (FMT) with kidney stone prevalence. Multivariable logistic regression models and restricted cubic spline analyses elucidated dose–response relationships, while stratified analyses explored population heterogeneity. Our investigation unveiled a compelling narrative of dietary protection: elevated DI‐GM scores conferred significant nephroprotection, with the most pronounced benefits observed in participants consuming microbiota‐supportive diets. Early meal initiation during the breakfast window (00:00–09:00) independently reduced stone risk compared to delayed feeding patterns. Remarkably, the convergence of optimal dietary quality with circadian‐aligned meal timing created a synergistic protective shield, yielding the most substantial risk reduction and suggesting that temporal nutrition strategies may amplify the benefits of gut microbiome modulation. This pioneering investigation establishes the foundational evidence for precision chrono‐nutritional interventions in kidney stone prevention. By harmonizing microbiome‐targeted dietary optimization with circadian meal timing, our findings illuminate a transformative pathway toward personalized therapeutic strategies that transcend conventional nutritional paradigms, offering new hope for millions affected by recurrent nephrolithiasis.

## Graphical abstract

This study reveals that higher Dietary Index for Gut Microbiota (DI‐GM ≥ 6) significantly reduces kidney stone risk by 22.7%. Early breakfast timing (00:00–09:00) provides additional protection. Combining optimal dietary quality with circadian‐aligned meal timing yields synergistic effects, reducing risk by 32% compared to a poor diet with delayed eating patterns.

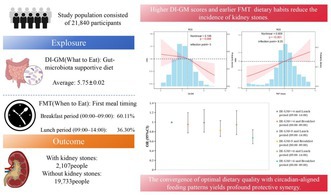

**Abbreviations**
BMI: body mass index
CDC: Centers for Disease Control and Prevention
CI: confidence interval
DI‐GM: Dietary index for gut microbiota
FMT: first meal timing
GM: gut microbiota
NCHS: National Center for Health Statistics
NHANES: National health and nutrition examination survey
OR: odds ratio
PIR: poverty income ratio
PSM: propensity score matching
RCS: restricted cubic spline
SCFAs: short‐chain fatty acids
SE: standard error
STROCSS: strengthening the reporting of cohort cross‐sectional and case–control studies in surgery
TRF: time‐restricted feeding
USDA: United States Department of Agriculture

## 1 Introduction

Kidney stones constitute a prevalent urological disorder demonstrating significant geographical heterogeneity in prevalence. Global epidemiological studies report considerable regional variation in adult kidney stone prevalence, typically ranging from 1% to 13%, with North American populations exhibiting rates between 7% and 13% (Peerapen and Thongboonkerd 2023; Sorokin et al. 2017). Furthermore, recurrence rates remain substantially elevated, with approximately 50% of patients experiencing recurrent episodes within 10 years of initial presentation (Chewcharat and Curhan 2021). In addition to the challenges in acute pain management, this condition can also lead to serious complications such as urinary tract infections, kidney damage and chronic kidney disease (Lindholt and Søgaard 2021). The resultant healthcare burden is considerable, with annual treatment expenditures in the United States exceeding $2 billion USD in 2000 (Lotan 2009). This substantial economic impact, coupled with high recurrence rates, underscores the critical need for effective prevention strategies through a deeper understanding of modifiable risk factors, particularly metabolic and dietary factors. Although kidney stones are increasingly regarded as a major public health issue related to metabolism, the understanding of the impact of diet, especially the role of the gut microbiota in stone formation remains limited (Al et al. 2023; Stanford et al. 2020). Comprehensive mechanistic investigations are consequently warranted to elucidate the complex pathophysiology underlying urolithiasis (Siener 2021).

Dietary habits constitute a pivotal modifiable risk factor in the pathogenesis of nephrolithiasis, operating primarily through two core dimensions: dietary composition (“what to eat”) and meal timing (“when to eat”). The gut microbiota (GM) exhibits established etiological links to nephrolithiasis and other pathologies like obesity, diabetes, and cardiovascular diseases (Crudele et al. 2023; Longo et al. 2023; Nesci et al. 2023). Reflecting this association, the Gut Microbiota Diet Index (DI‐GM) assesses dietary patterns as proxies for GM compositional states (Kase et al. 2024). DI‐GM explicitly categorizes foods into beneficial components such as whole grains, fermented dairy, legumes, and high‐fiber foods versus potentially detrimental components including red meat, processed meats, refined grains, and high‐fat diets. Higher DI‐GM scores correlate significantly with enhanced gut microbial diversity (Hou et al. 2023). This evidence positions dietary optimization as a viable GM modulation strategy.

Regarding meal timing, First Meal Timing (FMT) serves as a critical zeitgeber (Challet 2019). FMT entrains circadian oscillations in clock gene expression. These oscillations regulate diurnal rhythms governing digestive enzyme secretion, insulin sensitivity, and energy homeostasis. Consequently FMT exerts systemic metabolic effects (BaHammam and Pirzada 2023). Emerging evidence links FMT deviations (advancement or delay) to impaired glucose regulation, dyslipidemia, elevated oxidative stress biomarkers, and disrupted urinary concentration rhythms (Liu 2023). Preclinical studies confirm that Time‐Restricted Feeding (TRF) regimens stabilize FMT. TRF thereby reestablishes hepatic metabolic rhythms and enhances renal solute clearance efficiency, which may be related to the formation of urolithiasis (Yang et al. 2022).

This study aims to leverage data from the NHANES database to investigate the combined effects of DI‐GM and FMT on the incidence of kidney stones in U.S. adults. We seek to determine how dietary quality (assessed by DI‐GM) and circadian‐related feeding patterns (reflected by FMT) influence gut microbiota composition and their role in renal stone formation, ultimately providing scientific evidence for kidney stone prevention through dietary and chrono‐nutritional interventions.

## 2 Materials and Methods

### 2.1 Study Population

The National Health and Nutrition Examination Survey (NHANES), administered by the Centers for Disease Control and Prevention (CDC), is designed to evaluate the health status, dietary habits, and overall well‐being of the US population (National Center for Health Statistics (U.S.) 2013). NHANES gathers data through a nationally representative sample, utilizing a combination of surveys, clinical assessments, and laboratory analyses. The survey covers an extensive array of health‐related areas, encompassing chronic and infectious disease conditions, obesity, diabetes, cardiovascular disorders, dietary assessment, environmental exposure analysis, and oral health evaluation. The study protocols received ethical clearance from the National Center for Health Statistics (NCHS) Research Ethics Review Board under Protocols #2005–06 and #2011–17, with informed consent secured from all study participants. Additional information can be found on the official NHANES website: https://www.cdc.gov/nchs/nhanes/ (accessed on 30 October 2024).

**FIGURE 1:**
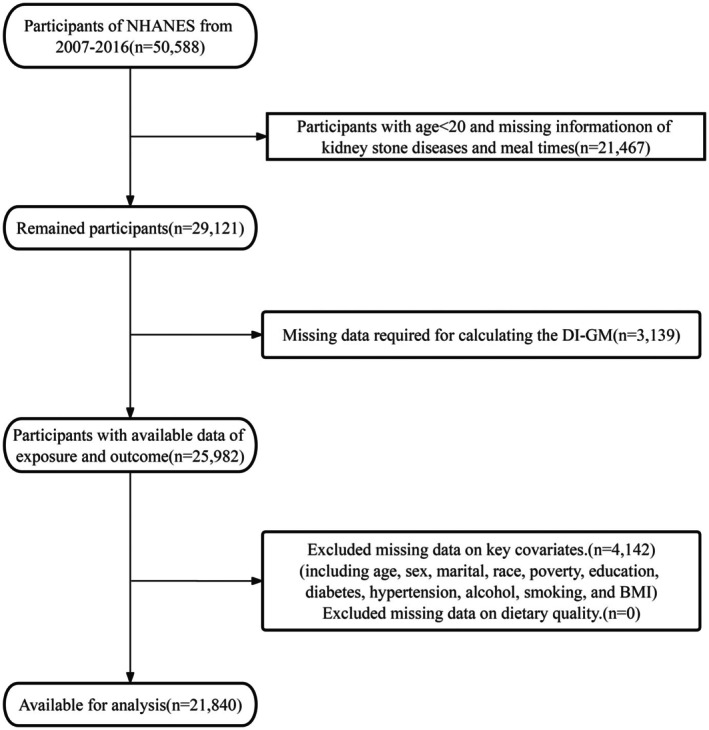
Flow chart of the screening of the NHANES 2007–2016 participants.

The present analysis utilized data from 50,588 participants enrolled in the National Health and Nutrition Examination Survey (NHANES, 2007–2016). We excluded 21,467 individuals due to age below 20 years or missing information regarding kidney stone history and first meal timing (FMT) parameters. Another 3139 participants were removed owing to inadequate data for computing the Dietary Index for Gut Microbiota (DI‐GM). Additionally, 4142 participants were excluded because of incomplete covariate information, including age, sex, race/ethnicity, marital status, poverty‐to‐income ratio, educational attainment, diabetes status, hypertension, alcohol consumption, smoking habits, or body mass index. Complete dietary quality information was available for all remaining participants, requiring no additional exclusions for this measure. The resulting study population consisted of 21,840 participants. The detailed inclusion and exclusion procedures are illustrated in Figure 1.

### 2.2 Study Variables

This study's principal endpoint was kidney stone occurrence, determined using self‐report methodology through the Health Status Questionnaire (HSQ) within the National Health and Nutrition Examination Survey (NHANES) framework. Study participants responded to the inquiry “Have you ever had a kidney stone?” using a dichotomous yes/no format. A positive response indicated previous kidney stone development. The validity of such self‐reported information has been established in prior studies (Duan et al. 2024).

NHANES employed a dual 24‐h dietary recall protocol for nutritional evaluation. The first recall session occurred during face‐to‐face interviews conducted at the Mobile Examination Center (MEC), followed by a secondary telephone‐based recall. These recall procedures captured detailed information regarding participants' food and beverage intake over the prior 24‐h period. Nutritional intake evaluation utilized the Dietary Index for Gut Microbiota (DI‐GM), developed from dietary recall information obtained within the NHANES 2007–2016 study population. The DI‐GM methodology, following the approach of Kase et al. (2024), encompasses 14 dietary elements or nutrients, each linked to either positive or negative influences on gut microbial community structure. Positive elements encompassed foods including avocado, broccoli, chickpeas, coffee, cranberries, fermented dairy products, dietary fiber, green tea (with tea type unspecified in NHANES), soybeans, and whole grains. Conversely, negative elements were defined by elevated consumption of red meat, processed meats, refined grains, and high‐fat dietary patterns (≥ 40% of total caloric intake). Individual component scoring utilized sex‐stratified median consumption levels: beneficial items received a score of 1 when consumed above median levels; otherwise 0; for detrimental items, scores of 0 were assigned when intake surpassed median or fat intake thresholds, with scores of 1 assigned otherwise. The total DI‐GM score, calculated by summing individual component values, represents overall dietary patterns that support gut microbiota wellness, where elevated scores indicate more beneficial nutritional practices.

A secondary dietary exposure examined in this study was the First Meal Timing (FMT), representing the temporal aspect of daily eating patterns (Sun et al. 2024). FMT was operationally defined as the chronological point of initial solid or liquid food consumption each day, extracted from the primary 24‐h dietary recall conducted at the Mobile Examination Center. The determination of FMT involved systematic identification of the earliest recorded food intake, followed by cross‐referencing with the corresponding food codes from the United States Department of Agriculture (USDA) food classification system integrated within the NHANES database (Supplementary USDA Food Code). For the purposes of this analysis, beverages including water, tea, alcoholic beverages, coffee, fruit juices, carbonated soft drinks, sports beverages, and energy drinks were excluded from FMT determination. While these liquid consumables may contain sugars, electrolytes, and various vitamins, they lack the comprehensive macronutrient profile including proteins, lipids, and dietary fiber necessary for sustained metabolic response and satiation. These beverages undergo rapid gastrointestinal absorption and elimination, thereby failing to provide the prolonged energy availability and appetite satisfaction characteristic of complete meals (Malik et al. 2006; Popkin et al. 2006). This approach ensures that FMT reflects meaningful meal timing patterns rather than incidental beverage consumption.

### 2.3 Covariates

Potential confounding factors were controlled through a comprehensive set of covariates, selected based on established clinical evidence and previous investigations (Duan et al. 2024; Zhang et al. 2024). The analysis incorporated data from the National Health and Nutrition Examination Survey (NHANES), encompassing sociodemographic parameters (age, sex, race/ethnicity stratified into Mexican, White, Black, and other categories), educational attainment (dichotomized at high school completion), marital status, and Poverty Income Ratio (PIR), body mass index (BMI). Smoking behavior was stratified according to lifetime cigarette consumption thresholds (Qiu et al. 2022): never smokers (< 100 cigarettes), current smokers (> 100 cigarettes), and former smokers (> 100 cigarettes with cessation).

Alcohol consumption patterns were evaluated through the Health Status Questionnaire's ALQ module, specifically utilizing item ALQ101 to assess 12‐month consumption history (Gao et al. 2023). Binary classification (yes/no) was employed to indicate historical alcohol use.

Diabetes mellitus status was determined through either physician diagnosis or documented use of antidiabetic medications, including insulin therapy. Similarly, hypertension was established through clinical diagnosis or prescribed antihypertensive medication.

Nutritional intake parameters—specifically energy (kcal), protein (g), carbohydrate (g), dietary fiber (g), total fat (g), total saturated fat (g), and cholesterol (mg) consumption—were extracted from the Individual Foods component of the Dietary Interview.

### 2.4 Statistical Analysis

Statistical procedures were executed utilizing the appropriate sampling weights from the National Health and Nutrition Examination Survey (NHANES), following the methodological standards established by the Centers for Disease Control and Prevention (CDC). Participant baseline characteristics were presented as mean values accompanied by standard errors (SEs) or as proportional distributions with corresponding SEs. To evaluate the relationship between the dietary index for gut microbiota (DI‐GM), first meal timing (FMT), and kidney stone prevalence, we implemented a series of three logistic regression approaches: a crude model without adjustments, a partially adjusted model, and a comprehensively adjusted model that incorporated pertinent confounding variables. The initial model (Model 1) remained unadjusted, whereas Model 2 incorporated adjustments for age, sex, race, marital status, poverty‐to‐income ratio (PIR), and educational attainment. Model 3 expanded upon Model 2 by including additional adjustments for body mass index (BMI), alcohol consumption patterns, hypertension status, diabetes mellitus, smoking habits, total energy intake, protein consumption, carbohydrate intake, dietary fiber consumption, total fat intake, total saturated fat intake, and cholesterol consumption.

We performed weighted multivariable logistic regression analyses to explore the associations between DI‐GM, FMT, and kidney stone prevalence, analyzing both DI‐GM and FMT as continuous measures and as categorical variables determined through threshold analysis outcomes. A restricted cubic spline (RCS) methodology utilizing three knots and incorporating threshold effects was implemented to examine potential nonlinear associations between the dietary index for gut microbiota (DI‐GM), first meal timing (FMT), and kidney stone occurrence.

For examining the combined effects of DI‐GM and FMT on kidney stone prevalence, study participants were stratified according to their DI‐GM and FMT levels, with multivariable logistic regression modeling applied to assess kidney stone risk estimates. Sensitivity testing encompassed subgroup analyses, multiple imputation procedures, and propensity score matching (PSM) techniques.

Statistical computations were performed using R software (version 4.4.2), incorporating the survey package (version 4.4–2) and the mediation package (version 4.5.0). Statistical significance was defined as *p* < 0.05. This investigation was performed in adherence to the strengthening the reporting of cohort, cross‐sectional, and case–control studies in surgery (STROCSS 2021) guidelines (Mathew et al. 2021).

## 3 Results

### 3.1 Baseline Characteristics

A total of 21,840 participants from the 2007 to 2016 NHANES cohort were analyzed, with baseline characteristics presented in Table 1. The mean age was 47.54 years ±0.29 years, with a balanced sex distribution (49.16% male, 50.84% female). The racial/ethnic composition comprised 7.98%, Mexican American, 10.64% non‐Hispanic Black, 69.21% non‐Hispanic White, and 12.17% other Hispanic participants.

**TABLE 1:** Characteristics of study participants (*N* = 21,840).

Characteristics	Total (*n* = 21,840)	Kidney stones		*p*
		No (*n* = 19,733)	Yes (*n* = 2107)	
Age (years)	47.54 ± 0.29	46.93 ± 0.31	53.23 ± 0.41	< 0.0001
Sex, %				
Female	50.84 (0.01)	51.51 (0.44)	44.63 (1.68)	< 0.001
Male	49.16 (0.01)	48.49 (0.44)	55.37 (1.68)	
Race/ethnicity, %				
Mexican American	7.98 (0.01)	8.18 (0.82)	6.12 (0.95)	< 0.0001
Non‐Hispanic White	69.21 (0.03)	68.18 (1.56)	78.75 (1.67)	
Non‐Hispanic Black	10.64 (0.01)	11.22 (0.83)	5.23 (0.64)	
Other Hispanic	12.17 (0.01)	12.41 (0.66)	9.90 (1.01)	
Marital status, %				
Married	54.13 (0.02)	53.19 (0.92)	62.90 (1.80)	< 0.0001
Never married	19.16 (0.01)	20.26 (0.88)	8.98 (0.98)	
Living with partner	7.92 (0.00)	8.09 (0.33)	6.37 (0.89)	
Other	18.78 (0.01)	18.46 (0.49)	21.76 (1.37)	
Education level, %				
≤ High school	37.67 (0.01)	37.57 (1.12)	38.59 (1.76)	0.48
> High school	62.33 (0.02)	62.43 (1.12)	61.41 (1.76)	
Family PIR, %	2.99 ± 0.04	2.98 ± 0.04	3.00 ± 0.06	0.83
Body mass index (kg/m^2^)	29.01 ± 0.09	28.84 ± 0.09	30.58 ± 0.23	< 0.0001
Smoking status, %				
Never smoker	54.44 (0.02)	55.12 (0.73)	48.07 (1.66)	< 0.0001
Former smoker	25.04 (0.01)	24.36 (0.60)	31.37 (1.46)	
Current smoker	20.52 (0.01)	20.51 (0.56)	20.56 (1.31)	
Drinking status, %				
Nondrinker	21.90 (0.01)	21.75 (0.78)	23.33 (1.41)	0.22
Drinker	78.10 (0.02)	78.25 (0.78)	76.67 (1.41)	
Hypertension, %				
No	67.67 (0.02)	69.14 (0.62)	53.99 (1.38)	< 0.0001
Yes	32.33 (0.01)	30.86 (0.62)	46.01 (1.38)	
Diabetes, %				
No	89.54 (0.03)	90.57 (0.33)	79.89 (1.04)	< 0.0001
Yes	10.46 (0.00)	9.43 (0.33)	20.11 (1.04)	
FMT (hours)	9.21 ± 0.04	9.21 ± 0.04	9.17 ± 0.07	0.59
Meal times category, %				
Breakfast period (00:00–09:00)	60.11 (0.02)	59.96 (0.82)	61.53 (1.50)	0.04
Lunch period (09:00–14:00)	36.30 (0.01)	36.30 (0.72)	36.23 (1.49)	
Evening Meal period (14:00–20:00)	3.44 (0.00)	3.58 (0.18)	2.09 (0.40)	
Late‐Night eating window (20:00–24:00)	0.16 (0.00)	0.16 (0.03)	0.15 (0.06)	
Energy level (kcal)	2150.92 ± 9.22	2157.05 ± 9.74	2093.76 ± 31.59	0.06
Protein intake (g)	83.20 ± 0.46	83.45 ± 0.47	80.87 ± 1.64	0.13
Carbohydrate intake (g)	255.83 ± 1.13	256.30 ± 1.23	251.44 ± 4.42	0.31
Dietary fber intake (g)	17.15 ± 0.18	17.20 ± 0.18	16.66 ± 0.41	0.18
Total fat intake (g)	82.62 ± 0.49	82.63 ± 0.48	82.49 ± 1.54	0.93
Total saturated fatty acids intake (g)	26.92 ± 0.19	26.94 ± 0.19	26.79 ± 0.55	0.79
Cholesterol intake (mg)	290.16 ± 2.46	289.01 ± 2.64	300.86 ± 7.77	0.16
DI‐GM, total	5.75 ± 0.02	5.76 ± 0.02	5.67 ± 0.05	0.03
Beneficial to gut microbiota	2.15 ± 0.02	2.16 ± 0.02	2.09 ± 0.04	0.08

Participants were stratified into two groups based on kidney stone history: stone formers (*n* = 2107, 9.6%) and nonstone formers (*n* = 19,733, 90.4%). Compared with nonstone formers, stone formers were older and had significantly higher BMI. Additionally, hypertension and diabetes prevalence were substantially elevated in stone formers (all *p* < 0.0001).

Regarding temporal dietary patterns, most participants initiated daily eating before 14:00, with the breakfast period (00:00–09:00) and lunch period (09:00–14:00) representing the primary feeding windows. Stone formers demonstrated modestly higher breakfast initiation rates but markedly reduced evening meal initiation (*p* < 0.05). No significant differences emerged in fasting duration or macronutrient intake between groups.

Stone formers exhibited a lower DI‐GM compared with nonstone formers (5.67 ± 0.05 vs. 5.76 ± 0.02, *p* = 0.03). The beneficial gut microbiota subcomponent showed a similar pattern but lacked statistical significance (*p* = 0.08).

### 3.2 Individual Associations Between DI‐GM or FMT and Kidney Stone

**FIGURE 2:**
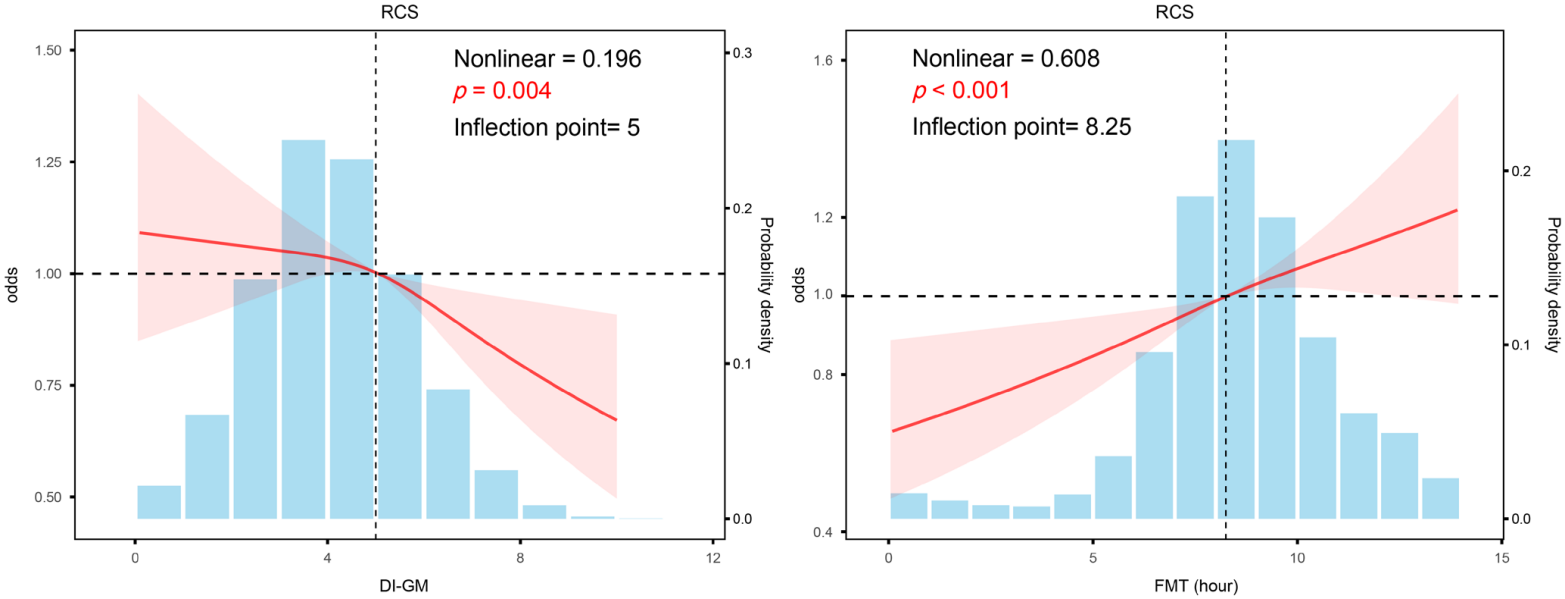
The restricted cubic spline analysis demonstrates the nonlinear relationships between DI‐GM, FMT and kidney stone risk, with adjustments made for age, sex, race, marital status, PIR, education level, BMI, smoking status, alcohol consumption, diabetes, hypertension, energy level, protein intake, carbohydrate intake, dietary fiber intake, total fat intake, total saturated fatty acid intake, and cholesterol intake. The 95% confidence intervals are depicted by the shaded regions.

As shown in Table 2, each unit increase in DI‐GM was associated with a 4.2% reduction in kidney stone risk in the fully adjusted model (Model III: OR = 0.958, 95% CI: 0.92–0.99, *p* = 0.022). The protective effect was most pronounced in the DI‐GM ≥ 6 subgroup (OR = 0.773, 95% CI: 0.671–0.890, *p* < 0.001). Beneficial gut microbiota profiles demonstrated significant inverse associations with kidney stone risk (OR = 0.934, 95% CI: 0.89–0.98, *p* = 0.014), while detrimental microbiota features showed no significant association. FMT analysis revealed a weak positive association for the continuous variable (OR = 1.026, 95% CI: 1.002–1.051, *p* = 0.041), with elevated risk observed in the 09:00–14:00 group (OR = 1.185, 95% CI: 1.032–1.361, *p* = 0.019). RCS analysis identified nonlinear associations between DI‐GM and kidney stone risk (P for nonlinear = 0.196, *p* = 0.004), with risk declining significantly beyond DI‐GM = 5. For FMT, risk increased beyond the 8.25‐h threshold. Notably, as shown in Figure 2, RCS analyses of FMT, the exposure window was truncated to 00:00–14:00, as most of the participants (60.11% breakfast [00:00–09:00] and 36.30% lunch [09:00–14:00] periods; see Table 1) initiated feeding within this interval. This temporal truncation enhanced analytical robustness by excluding extreme values beyond this biologically plausible range.

**TABLE 2:** Association between DI‐GM, FMT and kidney stones of the NHANES 2007–2016 participants.

Exposure	Model I^a^		Model II^b^		Model III^c^	
	OR (95% Cl)	*p*	OR (95% Cl)	*p*	OR (95% Cl)	*p*
DI‐GM	0.963 (0.93–0.99)	**0.023**	0.94 (0.91–0.97)	**< 0.001**	0.958 (0.92–0.99)	**0.022**
DI‐GM group						
0–4	1.000 (Reference)		1.000 (Reference)		1.000 (Reference)	
5	1.012 (0.874–1.172)	0.874	0.975 (0.84–1.133)	0.746	0.949 (0.817–1.103)	0.5
≥ 6	0.866 (0.754–0.994)	**0.045**	0.788 (0.68–0.913)	**0.002**	0.773 (0.671–0.890)	**< 0.001**
Beneficial to gut microbiota	0.958 (0.92–1)	0.057	0.924 (0.88–0.97)	**0.002**	0.934 (0.89–0.98)	**0.014**
Unfavorable to gut microbiota	0.979 (0.92–1.04)	0.481	0.98 (0.92–1.04)	0.504	0.995 (0.93–1.08)	0.872
FMT (continuous)	0.994 (0.974–1.015)	0.593	1.031 (1.006–1.056)	**0.016**	1.026 (1.002–1.051)	**0.041**
FMT (categorical)						
00:00–09:00	1.000 (Reference)		1.000 (Reference)		1.000 (Reference)	
09:00–14:00	0.973 (0.849–1.114)	0.69	1.195 (1.043–1.369)	**0.013**	1.185 (1.032–1.361)	**0.019**
14:00–20:00	0.568 (0.382–0.846)	0.07	0.887 (0.590–1.332)	0.564	0.813 (0.536–1.234)	0.335
20:00–24:00	0.929 (0.377–2.286)	0.872	1.464 (0.565–3.797)	0.435	1.176 (0.434–3.182)	0.751

*Note:* Bold values indicate that the overall data was statistically significant (*p* 〈 0.05).

^a^ Model I: no covariate adjustments were applied.

^b^ Model II: covariates included age, sex, race, marital status, PIR and education level.

^c^ Model III: covariates encompassed age, sex, race, marital status, PIR, education level, BMI, smoking status, alcohol consumption, diabetes, hypertension, energy level, protein intake, carbohydrate intake, dietary fiber intake, total fat intake, total saturated fatty acid intake, and cholesterol intake.

### 3.3 Joint Association of DI‐GM and FMT With Kidney Stone

In joint analyses of the Gut Microbiota Diet Index and first meal timing (FMT) (Table 3), individuals with the Gut Microbiota Diet Index ≥ 6 and breakfast‐period FMT (00:00–09:00) demonstrated the lowest risk of kidney stone incidence (OR 0.680, 95% CI: 0.550–0.842; *p* < 0.001), corresponding to a 32% risk reduction relative to the reference group (the Gut Microbiota Diet Index ≤ 4 and lunch‐period FMT [09:00–14:00]). This combined association exceeded the individual effects of the Gut Microbiota Diet Index ≥ 6 alone (OR 0.773, 95% CI: 0.671–0.890; *p* < 0.001) or breakfast‐period FMT alone (OR 0.853, 95% CI: 0.744–0.979; *p* = 0.027), indicating additive protective synergy. Intermediate risk reductions were observed for the Gut Microbiota Diet Index = 5 and breakfast‐period FMT (OR 0.839, 95% CI: 0.699–1.032; *p* = 0.989), whereas combinations involving lunch‐period FMT, such as the Gut Microbiota Diet Index ≥ 6 and lunch‐period FMT (OR 0.954, 95% CI: 0.734–1.241; *p* = 0.729), lacked statistical significance. Analysis of the data presented in Extended Data Figure 3 reveals that breakfast timing exerts a more pronounced influence on kidney stone incidence than the Gut Microbiota Diet Index.

**TABLE 3:** Joint association of DI‐GM and FMT with kidney stone among US adults aged 20 years or older.

Exposure	Model III^a^	
	OR (95% Cl)	*p*
DI‐GM alone		
DI‐GM ≤ 4	Ref	
DI‐GM = 5	0.949 (0.817–1.103)	0.5
DI‐GM ≥ 6	0.773 (0.671–0.890)	< 0.001
FMT alone		
Lunch period (09:00–14:00)	Ref	
Breakfast period (00:00–09:00)	0.853 (0.744–0.979)	0.027
Joint group		
DI‐GM ≤ 4 and Lunch period (09:00–14:00)	Ref	
DI‐GM ≤ 4 and Breakfast period (00:00–09:00)	0.963 (0.773–1.200)	0.739
DI‐GM = 5 and Lunch period (09:00–14:00)	0.998 (0.790–1.262)	0.189
DI‐GM = 5 and Breakfast period (00:00–09:00)	0.839 (0.699–1.032)	0.989
DI‐GM ≥ 6 and Lunch period (09:00–14:00)	0.954 (0.734–1.241)	0.729
DI‐GM ≥ 6 and Breakfast period (00:00–09:00)	0.680 (0.550–0.842)	< 0.001
P for trend	Ref	0.017

^a^ Model III: covariates encompassed age, sex, race, marital status, PIR, education level, BMI, smoking status, alcohol consumption, diabetes, hypertension, energy level, protein intake, carbohydrate intake, dietary fiber intake, total fat intake, total saturated fatty acid intake, and cholesterol intake.

**FIGURE 3:**
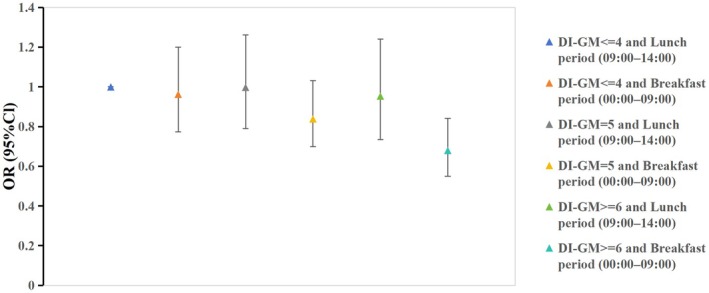
Joint association of DI‐GM and FMT with kidney stones among US adults aged 20 years or older: National Health and Nutrition Examination Survey, 2007–2016.

### 3.4 Sensitivity Analyses

Subgroup analyses stratified by sex, race, marital status, education level, drinking status, hypertension, diabetes, and smoking status revealed distinct associations between DI‐GM and FMT patterns and nephrolithiasis risk (Table S1). Females with DI‐GM ≥ 6 and 09:00–14:00 FMT exhibited significantly reduced risk (OR = 0.678, 95% CI 0.476–0.967, *p* = 0.032), contrasting with elevated risk in males with the same exposure profile (OR = 1.521, 95% CI 1.000–2.312, *p* = 0.050). Mexican Americans showed pronounced risk reduction (OR = 0.412, 95% CI 0.214–0.793, *p* = 0.009) when combining DI‐GM ≥ 6 with 00:00–09:00 FMT. Protective effects were consistently observed in individuals with high school education or less (OR = 0.686, 95% CI 0.499–0.943, *p* = 0.021) and alcohol consumers (OR = 0.670, 95% CI 0.521–0.861, *p* = 0.002) under DI‐GM ≥ 6 and 00:00–09:00 FMT conditions. Formal interaction tests demonstrated no significant heterogeneity across subgroups (all *p* interaction > 0.05), indicating stable population‐level associations. Multiple imputation analyses (Table S2) confirmed the stability of primary findings: the inverse association between DI‐GM and nephrolithiasis risk remained significant, particularly in the DI‐GM ≥ 6 subgroup. FMT analysis reveals that the rising trend of risk in the period from 09: 00 to 14: 00 is consistent in different models, which further supports the results of previous analysis.

## 4 Discussion

Using a nationally representative cross‐sectional design among U.S. adults, we investigated both the individual and combined relationships between the Dietary Index for Gut Microbiota (DI‐GM) and First Meal Timing (FMT) in relation to kidney stone prevalence. Higher DI‐GM scores demonstrated a significant inverse association with kidney stone risk (fully adjusted OR = 0.958 per unit increase; 95% CI: 0.92–0.99), corresponding to a 4.2% risk reduction per DI‐GM unit. Participants with DI‐GM scores ≥ 6 exhibited substantially lower odds of kidney stones (OR = 0.841; 95% CI: 0.725–0.975). Analysis of FMT revealed increased prevalence among individuals initiating their first meal during 09:00–14:00 (lunch period) versus 00:00–09:00 (breakfast period) (OR = 1.185; 95% CI: 1.032–1.361). Joint analysis identified a robust protective effect: subjects with both DI‐GM ≥ 6 and breakfast‐period FMT (00:00–09:00) manifested the lowest risk (OR = 0.680; 95% CI: 0.550–0.842), representing a 32% reduction relative to the reference group (DI‐GM ≤ 4 with lunch‐period FMT). To our knowledge, this constitutes the first comprehensive evaluation of both isolated and synergistic effects of a gut microbiota‐targeted dietary index and circadian‐aligned meal timing on nephrolithiasis in a nationally representative cohort.

Our findings demonstrate a robust inverse relationship between DI‐GM scores and the incidence of kidney stones, with optimal protective effects manifesting when scores reach ≥ 6. These results provide empirical support for the “gut‐kidney axis” hypothesis (Chen et al. 2019), wherein dietary elements associated with elevated DI‐GM scores—including fiber‐rich foods and fermented dairy products—exert regulatory effects on intestinal microbiome composition. This regulatory mechanism promotes the proliferation of beneficial bacterial strains, particularly Bifidobacteria, which possess oxalate‐degrading capabilities (Kase et al. 2024; Oliver et al. 2021). These microorganisms metabolize dietary fiber through fermentation processes, generating short‐chain fatty acids (SCFAs) including butyrate, acetate, and propionate (Scheppach 1994). These SCFAs can acidify urine, improving calcium and oxalate solubility, strengthening the intestinal barrier, regulating ion transport (including oxalate), and exerting anti‐inflammatory effects, collectively inhibiting the formation of calcium oxalate crystals (Al‐Harbi et al. 2018; Marzocco et al. 2018; Mitchell et al. 2019; Zhang et al. 2021). Conversely, low DI‐GM diets (e.g., rich in refined grains/animal fats) promote dysbiosis, leading to diminished SCFA production, accumulation of harmful metabolites (e.g., ammonia), and urine alkalinization—all of which elevate crystallization risk. Future metagenomics research is warranted to validate the mediating roles of specific microbial taxa and metabolic pathways within this diet‐microbiota‐kidney axis.

Late first meal timing (FMT beyond 09:00) fundamentally disrupts the physiological architecture of urinary chemistry rhythms. This temporal misalignment abolishes the natural diurnal variation in urine pH and compromises the rhythmic excretion patterns of citrate (Ratkalkar and Kleinman 2011; Siener 2021), a pivotal crystallization inhibitor. The metabolic consequences extend beyond urinary chemistry alterations. Late FMT triggers a cascade of insulin resistance, as demonstrated in controlled feeding studies where delayed energy intake consistently impairs glucose tolerance and increases HOMA‐IR values (Morris et al. 2015; Poggiogalle et al. 2018). This metabolic dysfunction manifests as elevated urinary calcium excretion accompanied by suppressed citrate output, creating a prolithogenic milieu (Taylor and Curhan 2006). The disruption of circadian meal timing further amplifies lithogenic risk through oxidative stress pathways. Compromised circadian antioxidant defenses, particularly the dysregulation of NRF2‐mediated protective mechanisms in BMAL1‐deficient states, intensify cellular oxidative burden (Manoogian and Panda 2017; Patel et al. 2016).

Our findings suggest potential synergistic protective effects between DI‐GM and FMT in kidney stone prevention, with individuals exhibiting DI‐GM ≥ 6 combined with breakfast‐period consumption (00:00–09:00) demonstrating a 32% risk reduction compared to the reference group. While these associations exceed those observed for single‐factor interventions, the underlying mechanisms warrant careful consideration. We propose that this apparent synergy may involve the temporal alignment of several interconnected biological processes.

The breakfast consumption window potentially coincides with enhanced dietary fiber processing efficiency. Morning intake may optimize circadian rhythms governing pancreatic enzyme secretion and bile acid circulation (Challet 2019), facilitating fermentation of DI‐GM‐recommended high‐fiber foods during periods of heightened gut microbiota metabolic activity. These SCFAs, particularly butyrate, may confer nephroprotection through urine acidification and anti‐inflammatory mechanisms (Jin et al. 2021).

Meal timing appears to influence microbial metabolic rhythms and their coordination with renal excretory processes. External zeitgebers, including feeding patterns, modulate host‐gut microbiome interactions through circadian regulation (Thaiss et al. 2014). Breakfast consumption may activate diurnal peaks in intestinal oxalate‐degrading bacterial activity (Hatch et al. 2006), potentially enhancing postprandial oxalate metabolism during periods when urinary oxalate excretion typically increases (Osswald and Hautmann 1979). Such temporal coordination could optimize the bioavailability of microbially derived protective compounds during critical crystallization windows, though direct evidence for this mechanism remains limited.

The coordination between circadian metabolic rhythms and gut microbiota‐host communication represents a third potential mechanism. Diurnal peaks in glomerular filtration rate, occurring approximately 2–4 h postmorning nutrient intake (Bloemen et al. 2009; Koopman et al. 1989), may create optimal clearance windows for microbially derived metabolites. Temporal alignment between SCFA production and enhanced renal function could facilitate urinary citrate excretion (Steenbeke et al. 2021), potentially inhibiting calcium oxalate crystallization. This mechanistic framework may explain why isolated high‐fiber consumption demonstrates limited protective effects when dissociated from circadian‐aligned timing patterns.

These findings provide a physiological foundation for integrated chrononutritional approaches targeting both dietary quality and temporal alignment in nephrolithiasis prevention. The cross‐sectional design of our study precludes definitive causal attribution, and these proposed mechanisms require validation through prospective investigations incorporating metagenomic profiling and detailed urinary biochemical analyses.

Our findings establish a mechanistic foundation for an integrated dietary strategy combining high‐fiber breakfast consumption with time‐restricted eating. This dual‐component approach mitigates inherent limitations of isolated interventions through concurrent engagement of microbial metabolic cycles and renal physiological rhythms. Although strengthened by the nationally representative NHANES cohort and comprehensive confounder adjustment, two principal limitations require acknowledgment. First, beverage exclusion in FMT operationalization may attenuate detection of hydration's potential lithoprotective effects. Second, the cross‐sectional design precludes definitive causal attribution regarding observed associations. Future prospective investigations should incorporate longitudinal metagenomic profiling and serial urinary biochemical analyses, with particular emphasis on dynamic oxalate‐to‐citrate molar ratios, to prioritize validation of the proposed pathophysiological pathways.

## 5 Conclusions

This investigation represents the first comprehensive examination of the synergistic relationship between gut microbiota‐targeted dietary patterns and circadian meal timing in kidney stone prevention. Our findings establish a compelling protective association between higher Dietary Index for Gut Microbiota scores and reduced nephrolithiasis risk, while demonstrating that early meal initiation during breakfast hours confers additional metabolic advantages. Most notably, the convergence of optimal dietary quality with circadian‐aligned feeding patterns yields profound protective synergy, surpassing the individual benefits of either intervention alone. These discoveries illuminate the intricate interplay between the gut‐kidney axis and chronobiological mechanisms in stone pathogenesis, offering a novel therapeutic paradigm that transcends conventional single‐factor approaches.

## Author Contributions

**Tianxiang Fu:** conceptualization, data curation, formal analysis, investigation, methodology, writing original draft. **Xuanhao Zhou:** conceptualization, data curation, formal analysis, investigation, methodology, writing – original draft. **Zhihong Chen:** conceptualization, data curation, formal analysis, investigation, methodology, writing – original draft. **Zhuoyan Huang:** validation, writing – review and editing. **Jingxian Li:** validation, writing – review and editing. **Wenqi Wu:** conceptualization, funding acquisition, project administration, resources, supervision, writing – review and editing. **Yiming Tang:** conceptualization, funding acquisition, project administration, resources, supervision, writing – review and editing. **Qiuyang Li:** conceptualization, funding acquisition, project administration, resources, supervision, writing – review and editing. All authors read and approved the final manuscript.

## Funding

This work was supported by the National Natural Science Foundation of China (Grant Number: 82270807), the Key Clinical Technique of Guangzhou, China (Grant Number: 2024CL‐ZD04), and GuangZhou Basic and Applied Basic Research Foundation (Grant Number: 2023A04J1866).

## Ethics Statement

The National Center for Health Statistics (NCHS) Research Ethics Review Board has approved the protocol. I confirm that all methods were performed in accordance with the relevant guidelines. All procedures were performed in accordance with the ethical standards laid down in the 1964 Declaration of Helsinki and its later amendments.

## Consent

All participants have provided informed written consent at the time of enrollment.

## Conflicts of Interest

The authors declare no conflicts of interest.

## Supporting information


**Table S1:** Stratified analyses of the associations between DI‐GM and FMT and the prevalence of kidney stone in NHANES 2007–2016.
**Table S2:** Association between DI‐GM, FMT and kidney stones of the NHANES 2007–2016 participants after multiple imputation.

## Data Availability

The datasets used and analyzed during the current study are available from the corresponding author on reasonable request.

## References

[fsn371297-bib-0001] Al, K. F. , B. R. Joris , B. A. Daisley , et al. 2023. “Multi‐Site Microbiota Alteration Is a Hallmark of Kidney Stone Formation.” Microbiome 11, no. 1: 263. 10.1186/s40168-023-01703-x. 38007438 PMC10675928

[fsn371297-bib-0002] Al‐Harbi, N. O. , A. Nadeem , S. F. Ahmad , et al. 2018. “Short Chain Fatty Acid, Acetate Ameliorates Sepsis‐Induced Acute Kidney Injury by Inhibition of NADPH Oxidase Signaling in T Cells.” International Immunopharmacology 58: 24–31. 10.1016/j.intimp.2018.02.023. 29544198

[fsn371297-bib-0003] BaHammam, A. S. , and A. Pirzada . 2023. “Timing Matters: The Interplay Between Early Mealtime, Circadian Rhythms, Gene Expression, Circadian Hormones, and Metabolism—A Narrative Review.” Clocks & Sleep 5, no. 3: 507–535. 10.3390/clockssleep5030034. 37754352 PMC10528427

[fsn371297-bib-0004] Bloemen, J. G. , K. Venema , M. C. Van De Poll , S. W. Olde Damink , W. A. Buurman , and C. H. Dejong . 2009. “Short Chain Fatty Acids Exchange Across the Gut and Liver in Humans Measured at Surgery.” Clinical Nutrition 28, no. 6: 657–661. 10.1016/j.clnu.2009.05.011. 19523724

[fsn371297-bib-0005] Challet, E. 2019. “The Circadian Regulation of Food Intake.” Nature Reviews Endocrinology 15, no. 7: 393–405. 10.1038/s41574-019-0210-x. 31073218

[fsn371297-bib-0006] Chen, Y.‐Y. , D.‐Q. Chen , L. Chen , et al. 2019. “Microbiome–Metabolome Reveals the Contribution of Gut–Kidney Axis on Kidney Disease.” Journal of Translational Medicine 17, no. 1: 5. 10.1186/s12967-018-1756-4. 30602367 PMC6317198

[fsn371297-bib-0007] Chewcharat, A. , and G. Curhan . 2021. “Trends in the Prevalence of Kidney Stones in the United States From 2007 to 2016.” Urolithiasis 49, no. 1: 27–39. 10.1007/s00240-020-01210-w. 32870387

[fsn371297-bib-0008] Crudele, L. , R. M. Gadaleta , M. Cariello , and A. Moschetta . 2023. “Gut Microbiota in the Pathogenesis and Therapeutic Approaches of Diabetes.” eBioMedicine 97: 104821. 10.1016/j.ebiom.2023.104821. 37804567 PMC10570704

[fsn371297-bib-0009] Duan, Q. , H. Huang , S. Zhang , et al. 2024. “Association Between Composite Dietary Antioxidant Index and Kidney Stone Prevalence in Adults: Data From National Health and Nutrition Examination Survey (NHANES, 2007–2018).” Frontiers in Nutrition 11: 1389714. 10.3389/fnut.2024.1389714. 38840700 PMC11150772

[fsn371297-bib-0010] Gao, M. , M. Liu , J. Chen , Z. Zhu , and H. Chen . 2023. “Association of Serum 25‐Hydroxyvitamin D Concentrations With All‐Cause Mortality Among Individuals With Kidney Stone Disease: The NHANES Database Prospective Cohort Study.” Frontiers in Endocrinology 14: 1207943. 10.3389/fendo.2023.1207943. 37854198 PMC10579890

[fsn371297-bib-0011] Hatch, M. , J. Cornelius , M. Allison , H. Sidhu , A. Peck , and R. W. Freel . 2006. “Oxalobacter sp. Reduces Urinary Oxalate Excretion by Promoting Enteric Oxalate Secretion.” Kidney International 69, no. 4: 691–698. 10.1038/sj.ki.5000162. 16518326

[fsn371297-bib-0012] Hou, J.‐L. , W.‐Y. Yang , Q. Zhang , et al. 2023. “Integration of Metabolomics and Transcriptomics to Reveal the Metabolic Characteristics of Exercise‐Improved Bone Mass.” Nutrients 15, no. 7: 1694. 10.3390/nu15071694. 37049535 PMC10097349

[fsn371297-bib-0013] Jin, X. , Z. Jian , X. Chen , et al. 2021. “Short Chain Fatty Acids Prevent Glyoxylate‐Induced Calcium Oxalate Stones by GPR43‐Dependent Immunomodulatory Mechanism.” Frontiers in Immunology 12: 729382. 10.3389/fimmu.2021.729382. 34675921 PMC8523925

[fsn371297-bib-0014] Kase, B. E. , A. D. Liese , J. Zhang , E. A. Murphy , L. Zhao , and S. E. Steck . 2024. “The Development and Evaluation of a Literature‐Based Dietary Index for Gut Microbiota.” Nutrients 16, no. 7: 1045. 10.3390/nu16071045. 38613077 PMC11013161

[fsn371297-bib-0015] Koopman, M. G. , G. C. M. Koomen , R. T. Krediet , E. A. M. De Moor , F. J. Hoek , and L. Arisz . 1989. “Circadian Rhythm of Glomerular Filtration Rate in Normal Individuals.” Clinical Science 77, no. 1: 105–111. 10.1042/cs0770105. 2667855

[fsn371297-bib-0016] Lindholt, J. S. , and R. Søgaard . 2021. “Why and When to Screen for Cardiovascular Disease in Healthy Individuals.” Heart 107, no. 12: 1010–1017. 10.1136/heartjnl-2019-316266. 33483351

[fsn371297-bib-0017] Liu, J. 2023. “The Effect of Early Time‐Restricted Eating vs Later Time‐Restricted Eating on Weight Loss and Metabolic Health.” Journal of Clinical Endocrinology and Metabolism 108, no. 7: 1824–1834. 10.1210/clinem/dgad036. 36702768

[fsn371297-bib-0018] Longo, S. , S. Rizza , and M. Federici . 2023. “Microbiota‐Gut‐Brain Axis: Relationships Among the Vagus Nerve, Gut Microbiota, Obesity, and Diabetes.” Acta Diabetologica 60, no. 8: 1007–1017. 10.1007/s00592-023-02088-x. 37058160 PMC10289935

[fsn371297-bib-0019] Lotan, Y. 2009. “Economics and Cost of Care of Stone Disease.” Advances in Chronic Kidney Disease 16, no. 1: 5–10. 10.1053/j.ackd.2008.10.002. 19095200

[fsn371297-bib-0020] Malik, V. S. , M. B. Schulze , and F. B. Hu . 2006. “Intake of Sugar‐Sweetened Beverages and Weight Gain: A Systematic Review.” American Journal of Clinical Nutrition 84, no. 2: 274–288. 10.1093/ajcn/84.1.274. 16895873 PMC3210834

[fsn371297-bib-0021] Manoogian, E. N. , and S. Panda . 2017. “Circadian Rhythms, Time‐Restricted Feeding, and Healthy Aging.” Ageing Research Reviews 39: 59–67. 10.1016/j.arr.2016.12.006. 28017879 PMC5814245

[fsn371297-bib-0022] Marzocco, S. , G. Fazeli , L. Di Micco , et al. 2018. “Supplementation of Short‐Chain Fatty Acid, Sodium Propionate, in Patients on Maintenance Hemodialysis: Beneficial Effects on Inflammatory Parameters and Gut‐Derived Uremic Toxins, A Pilot Study (PLAN Study).” Journal of Clinical Medicine 7, no. 10: 315. 10.3390/jcm7100315. 30274359 PMC6210519

[fsn371297-bib-0023] Mathew, G. , R. Agha , J. Albrecht , et al. 2021. “STROCSS 2021: Strengthening the Reporting of Cohort, Cross‐Sectional and Case‐Control Studies in Surgery.” International Journal of Surgery 96: 106165. 10.1016/j.ijsu.2021.106165. 34774726

[fsn371297-bib-0024] Mitchell, T. , P. Kumar , T. Reddy , et al. 2019. “Dietary Oxalate and Kidney Stone Formation.” American Journal of Physiology ‐ Renal Physiology 316, no. 3: F409–F413. 10.1152/ajprenal.00373.2018. 30566003 PMC6459305

[fsn371297-bib-0025] Morris, C. J. , J. N. Yang , J. I. Garcia , et al. 2015. “Endogenous Circadian System and Circadian Misalignment Impact Glucose Tolerance via Separate Mechanisms in Humans.” Proceedings of the National Academy of Sciences of the United States of America 112, no. 17: E2225–E2234. 10.1073/pnas.1418955112. 25870289 PMC4418873

[fsn371297-bib-0026] National Center for Health Statistics (U.S.) . 2013. National Health and Nutrition Examination Survey: Analytic Guidelines, 1999–2010. Hyattsville, Maryland U.S. Department of Health and Human Services, Centers for Disease Control and Prevention, National Center for Health Statistics.

[fsn371297-bib-0027] Nesci, A. , C. Carnuccio , V. Ruggieri , et al. 2023. “Gut Microbiota and Cardiovascular Disease: Evidence on the Metabolic and Inflammatory Background of a Complex Relationship.” International Journal of Molecular Sciences 24, no. 10: 9087. 10.3390/ijms24109087. 37240434 PMC10219307

[fsn371297-bib-0028] Oliver, A. , A. B. Chase , C. Weihe , et al. 2021. “High‐Fiber, Whole‐Food Dietary Intervention Alters the Human Gut Microbiome but Not Fecal Short‐Chain Fatty Acids.” MSystems 6, no. 2: e00115‐21. 10.1128/mSystems.00115-21. 33727392 PMC8546969

[fsn371297-bib-0029] Osswald, H. , and R. Hautmann . 1979. “Renal Elimination Kinetics and Plasma Half‐Life of Oxalate in Man.” Urologia Internationalis 34, no. 6: 440–450. 10.1159/000280294. 494445

[fsn371297-bib-0030] Patel, S. A. , A. Chaudhari , R. Gupta , N. Velingkaar , and R. V. Kondratov . 2016. “Circadian Clocks Govern Calorie Restriction–Mediated Life Span Extension Through BMAL1‐ and IGF‐1‐Dependent Mechanisms.” FASEB Journal 30, no. 4: 1634–1642. 10.1096/fj.15-282475. 26700733 PMC4799504

[fsn371297-bib-0031] Peerapen, P. , and V. Thongboonkerd . 2023. “Kidney Stone Prevention.” Advances in Nutrition 14, no. 3: 555–569. 10.1016/j.advnut.2023.03.002. 36906146 PMC10201681

[fsn371297-bib-0032] Poggiogalle, E. , H. Jamshed , and C. M. Peterson . 2018. “Circadian Regulation of Glucose, Lipid, and Energy Metabolism in Humans.” Metabolism, Clinical and Experimental 84: 11–27. 10.1016/j.metabol.2017.11.017. 29195759 PMC5995632

[fsn371297-bib-0033] Popkin, B. M. , L. E. Armstrong , G. M. Bray , B. Caballero , B. Frei , and W. C. Willett . 2006. “A New Proposed Guidance System for Beverage Consumption in the United States.” American Journal of Clinical Nutrition 83, no. 3: 529–542. 10.1093/ajcn.83.3.529. 16522898

[fsn371297-bib-0034] Qiu, Z. , X. Chen , T. Geng , et al. 2022. “Associations of Serum Carotenoids With Risk of Cardiovascular Mortality Among Individuals With Type 2 Diabetes: Results From NHANES.” Diabetes Care 45, no. 6: 1453–1461. 10.2337/dc21-2371. 35503926

[fsn371297-bib-0035] Ratkalkar, V. N. , and J. G. Kleinman . 2011. “Mechanisms of Stone Formation.” Clinical Reviews in Bone and Mineral Metabolism 9, no. 3–4: 187–197. 10.1007/s12018-011-9104-8. 22229020 PMC3252394

[fsn371297-bib-0036] Scheppach, W. 1994. “Effects of Short Chain Fatty Acids on Gut Morphology and Function.” Gut 35, no. 1 Suppl: S35–S38. 10.1136/gut.35.1_Suppl.S35. 8125387 PMC1378144

[fsn371297-bib-0037] Siener, R. 2021. “Nutrition and Kidney Stone Disease.” Nutrients 13, no. 6: 1917. 10.3390/nu13061917. 34204863 PMC8229448

[fsn371297-bib-0038] Sorokin, I. , C. Mamoulakis , K. Miyazawa , A. Rodgers , J. Talati , and Y. Lotan . 2017. “Epidemiology of Stone Disease Across the World.” World Journal of Urology 35, no. 9: 1301–1320. 10.1007/s00345-017-2008-6. 28213860

[fsn371297-bib-0039] Stanford, J. , K. Charlton , A. Stefoska‐Needham , R. Ibrahim , and K. Lambert . 2020. “The Gut Microbiota Profile of Adults With Kidney Disease and Kidney Stones: A Systematic Review of the Literature.” BMC Nephrology 21, no. 1: 215. 10.1186/s12882-020-01805-w. 32503496 PMC7275316

[fsn371297-bib-0040] Steenbeke, M. , S. Valkenburg , T. Gryp , et al. 2021. “Gut Microbiota and Their Derived Metabolites, a Search for Potential Targets to Limit Accumulation of Protein‐Bound Uremic Toxins in Chronic Kidney Disease.” Toxins 13, no. 11: 809. 10.3390/toxins13110809. 34822593 PMC8625482

[fsn371297-bib-0041] Sun, T. , L. Zhang , Y. Lu , et al. 2024. “Non‐Linear Relationship Between the First Meal Time of the Day and Gallstone Incidence in American Adults: A Population‐Based Cross‐Sectional Study.” Frontiers in Nutrition 11: 1521707. 10.3389/fnut.2024.1521707. 39737153 PMC11684388

[fsn371297-bib-0042] Taylor, E. N. , and G. C. Curhan . 2006. “Body Size and 24‐Hour Urine Composition.” American Journal of Kidney Diseases 48, no. 6: 905–915. 10.1053/j.ajkd.2006.09.004. 17162145

[fsn371297-bib-0043] Thaiss, C. A. , D. Zeevi , M. Levy , et al. 2014. “Transkingdom Control of Microbiota Diurnal Oscillations Promotes Metabolic Homeostasis.” Cell 159, no. 3: 514–529. 10.1016/j.cell.2014.09.048. 25417104

[fsn371297-bib-0044] Yang, M. , W. Chen , L. He , D. Liu , L. Zhao , and X. Wang . 2022. “Intermittent Fasting—A Healthy Dietary Pattern for Diabetic Nephropathy.” Nutrients 14, no. 19: 3995. 10.3390/nu14193995. 36235648 PMC9571963

[fsn371297-bib-0045] Zhang, X. , Y. Tong , X. Lyu , J. Wang , Y. Wang , and R. Yang . 2021. “Prevention and Alleviation of Dextran Sulfate Sodium Salt‐Induced Inflammatory Bowel Disease in Mice With *Bacillus subtilis* ‐Fermented Milk via Inhibition of the Inflammatory Responses and Regulation of the Intestinal Flora.” Frontiers in Microbiology 11: 622354. 10.3389/fmicb.2020.622354. 33519783 PMC7845695

[fsn371297-bib-0046] Zhang, X. , Q. Yang , J. Huang , H. Lin , N. Luo , and H. Tang . 2024. “Association of the Newly Proposed Dietary Index for Gut Microbiota and Depression: The Mediation Effect of Phenotypic Age and Body Mass Index.” European Archives of Psychiatry and Clinical Neuroscience 275: 1037–1048. 10.1007/s00406-024-01912-x. 39375215

