# Supplementary material for: Chrono‐Synergistic Nutrition: Gut Microbiota–Targeted Diets and Circadian‐Aligned First Meal Timing Confer Robust Protection Against Kidney Stone Formation
Source: Food Sci Nutr. 2025 Dec 15;13(12):e71297. doi: 10.1002/fsn3.71297 (PMC12705486; doi:10.1002/fsn3.71297)
Supplement: Supplementary file 1 — Table S1: Stratified analyses of the associations between DI‐GM and FMT and the prevalence of kidney stone in NHANES 2007–2016. Table S2: Association between DI‐GM, FMT and kidney stones of the NHANES 2007–2016 participants after multiple imputation. [file FSN3-13-e71297-s001.docx]

**Supplementary Materials Files**

**Weighted Analysis**

Weighted analysis is a statistical technique that assigns varying weights to individual observations in a sample, reflecting their relative importance and representativeness within the broader dataset. This method enhances the precision and efficiency of estimates, offering deeper insights into the characteristics of the population and the relationships among variables. By adjusting for sample weights, weighted analysis corrects for potential biases in estimation, thus improving the accuracy of the results. Moreover, by tailoring weights according to the importance of observations, the technique ensures that the sample accurately represents the target population, leading to more reliable population-level estimates. For detailed guidance on NHANES weighting methodology, we refer to the NHANES website. In line with these protocols, we incorporated both the complex sampling design and the sample weights from the mobile examination centers into our analysis, ensuring that the data reflect the diversity and scale of the U.S. adult population.

**Multiple Imputation**

Multiple imputation is a well-established and widely applied statistical technique for addressing missing data, with notable utility in fields such as epidemiology, social sciences, and clinical research. The method involves generating several plausible datasets through imputation, conducting separate analyses on each, and subsequently pooling the results to produce valid estimates along with measures of uncertainty. This approach helps to reduce the biases associated with missing data, ensuring the robustness and representativeness of the findings. In this study, we employed the multiple imputation by chained equations (MICE) method, a flexible technique that imputes missing values iteratively for each variable using regression models, conditioned on the observed values of other variables in the dataset.

**Supplementary Table**

**Supplementary Table 1. Stratified analyses of the associations between DI-GM and FMT and the prevalence of kidney stone in NHANES 2007–2016.**

**Supplementary Table 2. Association between DI-GM, FMT and kidney stones of the NHANES 2007–2016 participants after multiple imputation.**

| **Supplementary Table 1.** Stratified analyses of the associations between DI-GM and FMT and the prevalence of kidney stone in NHANES 2007–2016. | | | | | | | | | | | | | |
| --- | --- | --- | --- | --- | --- | --- | --- | --- | --- | --- | --- | --- | --- |
| Subgroup | DIGM≤4 and (09:00-14:00) | DIGM≤4 and (00:00-09:00) | | DIGM=5 and (09:00-14:00) | | DIGM=5 and (00:00-09:00) | | DIGM≥6 and (09:00-14:00) | | DIGM≥6 and (00:00-09:00) | |  |  |
|  |  | OR (95%Cl) | *P* value | OR (95%Cl) | *P* value | OR (95%Cl) | *P* value | OR (95%Cl) | *P* value | OR (95%Cl) | *P* value | *P* for trend(character 2 integer) | *P* for interaction |
| Sex |  |  |  |  |  |  |  |  |  |  |  |  | 0.081 |
| Female | ref | 0.830(0.619,1.113) | 0.209 | 0.957(0.689,1.331) | 0.792 | 0.905(0.653,1.255) | 0.544 | 0.678(0.476,0.967) | **0.032** | 0.709(0.530,0.949) | **0.022** | **0.085** |  |
| Male | ref | 1.091(0.819,1.454) | 0.547 | 1.148(0.761,1.731) | 0.504 | 0.965(0.664,1.404) | 0.852 | 1.521(1.000,2.312) | **0.050** | 0.791(0.575,1.090) | 0.149 | 0.757 |  |
| Race/ethnicity |  |  |  |  |  |  |  |  |  |  |  |  | 0.17 |
| Mexican American | ref | 0.834(0.501,1.388) | 0.477 | 0.867(0.462,1.627) | 0.650 | 0.892(0.521,1.529) | 0.672 | 0.914(0.537,1.558) | 0.737 | 0.412(0.214,0.793) | **0.009** | 0.091 |  |
| Non-Hispanic White | ref | 0.921(0.694,1.222) | 0.563 | 1.054(0.771,1.440) | 0.738 | 0.983(0.724,1.335) | 0.912 | 1.115(0.801,1.553) | 0.514 | 0.777(0.589,1.026) | 0.074 | 0.239 |  |
| Non-Hispanic Black | ref | 1.019(0.688,1.508) | 0.925 | 0.928(0.531,1.621) | 0.790 | 0.891(0.486,1.634) | 0.705 | 0.889(0.556,1.421) | 0.616 | 0.668(0.389,1.147) | 0.140 | 0.323 |  |
| Other Hispanic | ref | 1.301(0.871,1.944) | 0.194 | 1.167(0.664,2.051) | 0.586 | 0.560(0.328,0.955) | **0.034** | 0.621(0.355,1.089) | 0.095 | 0.662(0.400,1.096) | 0.107 | 0.638 |  |
| Marital status |  |  |  |  |  |  |  |  |  |  |  |  | 0.259 |
| Married | ref | 0.864(0.668,1.118) | 0.263 | 0.952(0.680,1.333) | 0.772 | 0.757(0.561,1.022) | 0.068 | 1.086(0.756,1.559) | 0.651 | 0.626(0.464,0.844) | **0.003** | 0.119 |  |
| Never married | ref | 1.166(0.591,2.298) | 0.653 | 1.295(0.692,2.425) | 0.413 | 0.822(0.329,2.056) | 0.671 | 1.006(0.440,2.305) | 0.988 | 1.523(0.755,3.073) | 0.236 | 0.202 |  |
| Living with partner | ref | 0.848(0.365, 1.968) | 0.696 | 0.494(0.185, 1.321) | 0.157 | 0.741(0.301, 1.825) | 0.508 | 0.740(0.288, 1.901) | 0.526 | 0.656(0.247, 1.742) | 0.392 | 0.278 |  |
| Other | ref | 1.143(0.740,1.765) | 0.541 | 1.483(0.796,2.763) | 0.211 | 1.709(1.047,2.788) | **0.033** | 1.020(0.595,1.748) | 0.942 | 0.933(0.581,1.498) | 0.771 | 0.645 |  |
| Education level |  |  |  |  |  |  |  |  |  |  |  |  | 0.094 |
| ≤ High school | ref | 0.798(0.587,1.086) | 0.148 | 0.961(0.684,1.350) | 0.814 | 0.914(0.641,1.302) | 0.613 | 1.220(0.816,1.824) | 0.327 | 0.885(0.598,1.308) | 0.534 | 0.872 |  |
| > High school | ref | 1.080(0.812,1.435) | 0.592 | 1.100(0.808,1.499) | 0.539 | 0.946(0.693,1.291) | 0.722 | 0.953(0.686,1.323) | 0.769 | 0.686(0.499,0.943) | **0.021** | 0.092 |  |
| Drinking status |  |  |  |  |  |  |  |  |  |  |  |  | 0.34 |
| Nondrinker | ref | 0.995(0.683,1.449) | 0.979 | 1.240(0.773,1.990) | 0.366 | 0.845(0.564,1.266) | 0.409 | 0.951(0.526,1.719) | 0.866 | 1.012(0.668,1.533) | 0.953 | 0.374 |  |
| Drinker | ref | 0.934(0.711,1.227) | 0.617 | 0.982(0.734,1.315) | 0.903 | 0.950(0.709,1.273) | 0.727 | 1.032(0.777,1.371) | 0.826 | 0.670(0.521,0.861) | **0.002** | **0.025** |  |
| Hypertension |  |  |  |  |  |  |  |  |  |  |  |  | 0.173 |
| No | ref | 0.941(0.713,1.242) | 0.663 | 0.899(0.635,1.272) | 0.541 | 1.074(0.788,1.466) | 0.645 | 1.038(0.740,1.457) | 0.825 | 0.730(0.538,0.990) | **0.043** | **0.035** |  |
| Yes | ref | 0.967(0.707,1.323) | 0.832 | 1.254(0.861,1.826) | 0.233 | 0.758(0.535,1.072) | 0.115 | 1.002(0.711,1.411) | 0.992 | 0.748(0.555,1.007) | 0.056 | 0.952 |  |
| Diabetes |  |  |  |  |  |  |  |  |  |  |  |  | 0.66 |
| No | ref | 0.985(0.769,1.260) | 0.900 | 1.024(0.775,1.353) | 0.865 | 0.966(0.758,1.231) | 0.777 | 0.973(0.747,1.268) | 0.837 | 0.760(0.597,0.968) | **0.027** | 0.133 |  |
| Yes | ref | 0.833(0.555,1.250) | 0.371 | 1.165(0.668,2.032) | 0.585 | 0.755(0.412,1.383) | 0.356 | 1.296(0.755,2.225) | 0.341 | 0.664(0.378,1.166) | 0.151 | 0.849 |  |
| Smoking status |  |  |  |  |  |  |  |  |  |  |  |  | 0.654 |
| Never smoker | ref | 0.894(0.669,1.196) | 0.445 | 1.052(0.753,1.468) | 0.763 | 0.835(0.601,1.162) | 0.280 | 0.956(0.675,1.354) | 0.797 | 0.751(0.540,1.045) | 0.088 | 0.485 |  |
| Former smoker | ref | 0.919(0.629,1.342) | 0.657 | 0.727(0.435,1.214) | 0.219 | 0.804(0.514,1.257) | 0.333 | 1.038(0.652,1.651) | 0.874 | 0.607(0.395,0.934) | **0.024** | **0.045** |  |
| Current smoker | ref | 1.160(0.776,1.732) | 0.463 | 1.578(1.008,2.471) | **0.046** | 1.400(0.890,2.201) | 0.142 | 1.139(0.674,1.926) | 0.621 | 1.012(0.604,1.697) | 0.963 | 0.622 |  |
| Abbreviations: NHANES: National Health and Nutrition Examination Survey; OR: odds ratio: CI: confidence interval. | | | | | | | | | | | | | |
| Notes: sex, race, marital status, education level, drinking status, hypertension, diabetes, and smoking status were adjusted. | | | | | | | | | | | | | |

| **Supplementary Table 2.** Association between DI-GM, FMT and kidney stones of the NHANES 2007–2016 participants after multiple imputation. | | | | | | | | |
| --- | --- | --- | --- | --- | --- | --- | --- | --- |
| Exposure | Model Ia | |  | Model IIb | |  | Model IIIc | |
|  | OR (95%Cl) | *P* value |  | OR (95%Cl) | *P* value |  | OR (95%Cl) | *P* value |
| DI-GM | 0.986 (0.960 - 1.013) | 0.316 |  | 0.960 (0.934 - 0.987) | **0.005** |  | 0.975 (0.947 - 1.003) | 0.08 |
| DI-GM group |  |  |  |  |  |  |  |  |
| 0–4 | 1.000 (Reference) |  |  | 1.000 (Reference) |  |  | 1.000 (Reference) |  |
| 5 | 1.005 (0.883 - 1.144) | 0.939 |  | 0.948 (0.829 - 1.084) | 0.439 |  | 0.977 (0.855 - 1.116) | 0.732 |
| ≥6 | 0.914 (0.807 - 1.036) | 0.164 |  | 0.806 (0.703 - 0.924) | **0.003** |  | 0.858 (0.744 - 0.989) | **0.039** |
| Beneficial to gut microbiota | 0.981 (0.943 - 1.020) | 0.344 |  | 0.939 (0.900 - 0.980) | **0.005** |  | 0.948 (0.908 - 0.989) | **0.016** |
| Unfavorable to gut microbiota | 0.994 (0.953 - 1.037) | 0.782 |  | 0.990 (0.948 - 1.034) | 0.64 |  | 1.015 (0.972 - 1.060) | 0.503 |
|  |  |  |  |  |  |  |  |  |
| FMT (continuous) | 0.998 (0.993 - 1.003) | 0.483 |  | 1.007 (1.001 - 1.013) | **0.026** |  | 1.006 (1.000 - 1.012) | **0.064** |
| FMT (categorical) |  |  |  |  |  |  |  |  |
| 00:00-09:00 | 1.000 (Reference) |  |  | 1.000 (Reference) |  |  | 1.000 (Reference) |  |
| 09:00-14:00 | 0.988 (0.866 - 1.127) | 0.854 |  | 1.213 (1.062 - 1.384) | **0.006** |  | 1.202 (1.052 - 1.375) | **0.009** |
| 14:00-20:00 | 0.624 (0.436 - 0.894) | **0.012** |  | 0.946 (0.663 - 1.349) | 0.758 |  | 0.872 (0.607 - 1.253) | 0.462 |
| 20:00-24:00 | 0.944 (0.416 - 2.143) | 0.891 |  | 1.561 (0.661 - 3.691) | 0.314 |  | 1.329 (0.537 - 3.290) | 0.541 |
| aModel I: adjusted for none. | | | | | | | | |
| bModel II: adjusted for age, sex,race, marital status, PIR and education level. | | | | | | | | |
| cModel III: adjusted for age, sex, race, marital status, PIR, education level, BMI, smoking status, alcohol consumption, diabetes, hypertension, energy level, protein intake, carbohydrate intake, dietary fiber intake, total fat intake, total saturated fatty acid intake, and cholesterol intake. | | | | | | | | |
|  |  |  |  |  |  |  |  |  |
